# A WKYMVm-Containing Combination Elicits Potent Anti-Tumor Activity in Heterotopic Cancer Animal Model

**DOI:** 10.1371/journal.pone.0030522

**Published:** 2012-01-25

**Authors:** Sang Doo Kim, Ha Young Lee, Jae Woong Shim, Hak Jung Kim, Suk-Hwan Baek, Brian A. Zabel, Yoe-Sik Bae

**Affiliations:** 1 Department of Biological Sciences, Sungkyunkwan University, Suwon, South Korea; 2 Mitochondria Hub Regulation Center, Dong-A University, Busan, South Korea; 3 Department of Biochemistry and Molecular Biology, College of Medicine, Yeungnam University, Daegu, Korea; 4 Palo Alto Institute for Research and Education, Veterans Affairs Hospital, Palo Alto, California, United States of America; Technische Universität München, Germany

## Abstract

The development of efficient anti-cancer therapy has been a topic of intense interest for several decades. Combined administration of certain molecules and immune cells has been shown to be an effective form of anti-cancer therapy. Here, we examined the effects of administering an immune stimulating peptide (WKYMVm), 5-fluoro-uracil (5-FU), and mature dendritic cells (mDCs) against heterotopic cancer animal model. Administration of the triple combination strongly reduced tumor volume in CT-26-inoculated heterotopic cancer animal model. The induced anti-tumor activity was well correlated with FAS expression, caspase-3 activation, and cancer cell apoptosis. The triple combination treatment caused recruitment of CD8 T lymphocytes and natural killer (NK) cells into the tumor. The production of two cytokines, IFN-γ and IL-12, were strongly stimulated by administration of the triple combination. Depletion of CD8 T lymphocytes or NK cells by administration of anti-CD8 or anti-asialoGM1 antibody inhibited the anti-tumor activity and cytokine production of the triple combination. The triple combination strongly inhibited metastasis of colon cancer cells in a heterotopic cancer animal model as well as in a metastatic cancer animal model, and enhanced the survival rate of the mice model. Adoptive transfer of CD8 T lymphocytes and NK cells further increased the survival rate. Taken together, we suggest that the use of triple combination therapy of WKYMVm, 5-FU, and mDCs may have implications in solid tumor and metastasis treatment.

## Introduction

Development of anti-cancer therapy has been an important issue for several decades [1], [2]. Treatment with anti-cancer agents is one of the most widely utilized modes of anti-tumor therapy. Various anti-cancer agents have been developed, including 5-fluoro-uracil (5-FU) [3]. Mechanistically, anti-cancer agents have been reported to cause apoptosis of cancer cells, which induces the initiation of anti-cancer immune responses [4]. In the host immune system, dendritic cells (DCs) appear to play a key role in anti-tumor activity. DCs recognize and uptake tumor antigens, and present the processed antigens on major histocompatibility complex molecules [5], [6], [7]. Thus, DCs can stimulate T lymphocytes, resulting in cytotoxic T lymphocyte activity. DCs also secrete several cytokines which are important in efficient anti-tumor activity [7].

WKYMVm was identified as an immune-stimulating synthetic peptide from a peptide library screening [8], [9]. WKYMVm stimulates leukocytic cells such as monocytes, neutrophils, natural killer (NK) cells, and DCs [9], [10], [11], [12]. Because of monocyte and neutrophil stimulation, chemotactic migration, superoxide anion production, and the production of certain inflammatory mediators such as leukotriene B_4_ is induced by WKYMVm [9], [13], [14]. NK cell stimulation with WKYMVm results in cytolytic activity and chemotactic migration [11]. The peptide stimulated chemotactic migration of DCs [12]. Three members of the formyl peptide receptor (FPR) family have been reported to be cognitive cell surface receptors for WKYMVm in humans [15], [16]. Mouse FPR has also been reported to act as a WKYMVm receptor [17]. Although WKYMVm has been reported to stimulate leukocytes which play important roles against cancer antigens, little is known about the role of WKYMVm in anti-cancer activity.

Combined administration of certain molecules can induce effective anti-cancer activity. Although various anti-cancer agents or anti-cancer therapies have been reported, development of new anti-cancer therapies which are effective and specific with low toxicity is still necessary. In this study we investigated the therapeutic activity of WKYMVm when it was administrated with an anti-cancer agent (5-FU) and a natural vaccine adjuvant (mature DCs, mDCs). The mechanism of action of the triple combination therapy was also characterized.

## Results

### Combined administration of WKYMVm, 5-FU, and mDCs causes anti-tumor activity in heterotopic cancer animal model

**Figure 1:**
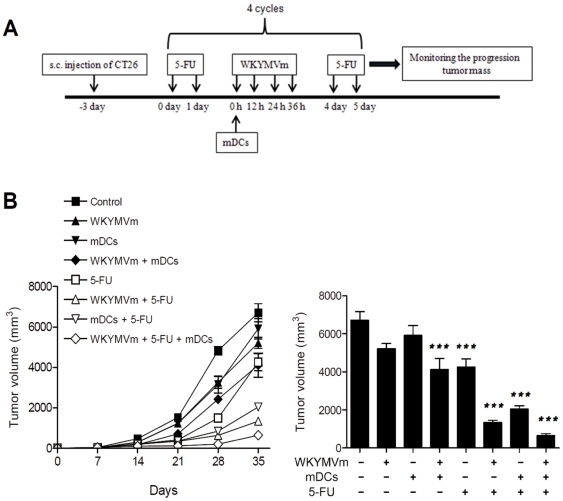
Anti-tumor activity of WKYMVm, 5-FU, and mature DCs in a heterotopic cancer animal model. (A) Protocol for the study of anti-tumor activity of WKYMVm, 5-FU, and mDCs. (B) The triple combination of WKYMVm, 5-FU, and mDCs has the most potent anti-tumor activity. CT-26 cells (5×10^5^ cells in 100 µl of PBS) were injected s.c. into the right flank of Balb/c mice (n = 8) on day −3. The mice were treated according to the protocol in (A). Tumor volume was measured and the data are shown as the mean ± SEM (n = 8). ^***^, *P*<0.001 compared with the control.

The putative anti-tumor activity of WKYMVm, 5-FU, or mDCs was examined. WKYMVm, 5-FU, or mDCs were first administered singly. For example, as shown in Fig. 1A, WKYMVm (100 µg/head) was injected four times at 12 h intervals. Single administration caused a slight decrease in tumor volume (Fig. 1B). When the agents were tested in pairs (WKYMVm+5-FU; WKYMVm+mDCs; mDCs+5-FU) against an animal model, anti-tumor activity was enhanced (Fig. 1B). The presence of 5-FU appeared to further potentiate anti-tumor activity (Fig. 1B, 5-FU+WKYMVm and 5-FU+mDCs). Moreover, when the triple combination (WKYMVm+5-FU+mDCs) was administered to the heterotopic cancer animal model, the most potent anti-tumor activity was observed (Fig. 1B).

### Combined administration of WKYMVm, 5-FU, and mDCs elicits tumor apoptosis

**Figure 2:**
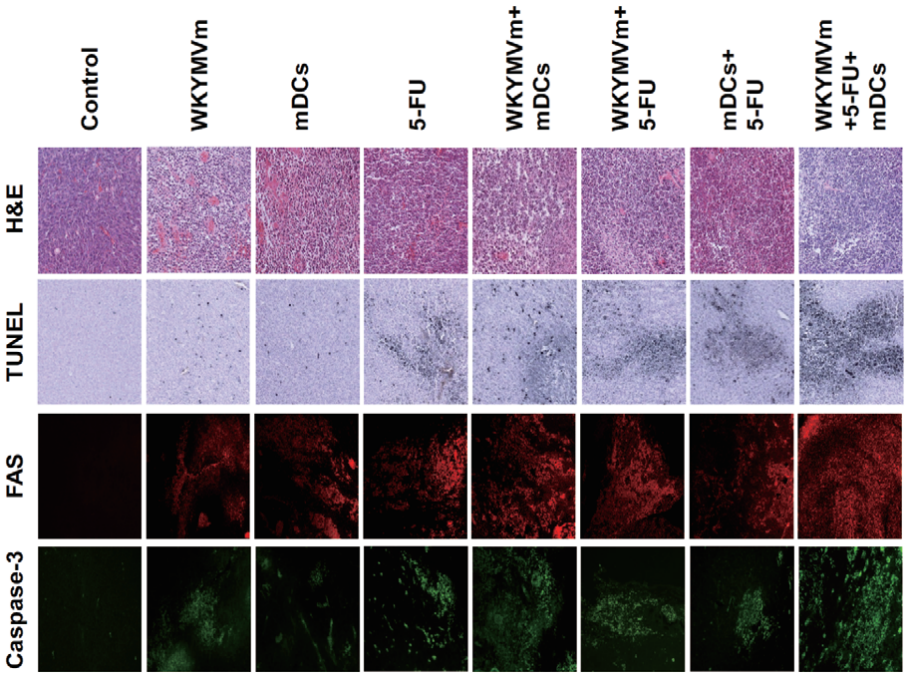
Administration of WKYMVm, 5-FU, and mDCs causes tumor cell apoptosis in a heterotopic cancer animal model. Balb/c mice were inoculated with CT-26 cells and treated with WKYMVm, 5-FU, and mDCs according to the protocol in Fig. 1A. Mice were euthanized 42 days after tumor inoculation and tumors were surgically excised, and processed for hematoxylin and eosin staining, immunohistochemistry for FAS staining, caspase-3 staining, and TUNEL staining, as described in Materials and Methods. The data presented is representative of three independent experiments.

Since tumor apoptosis is closely related to anti-tumor activity, the effect of each administration on tumor apoptosis was measured. As shown in Fig. 2, single administration of WKYMVm, 5-FU, or mDCs induced low levels of tumor cell death, and double combinations showed further increase in cell death, with the triple combination resulting in dramatically increased tumor cell death in the heterotopic cancer animal model (Fig. 2). The TUNEL-positive cells coincided with FAS expression, indicating that the tumor cell death observed is due to apoptosis (Fig. 2). Caspase-3 activity upon treatment was determined by immunofluorescence staining using anti-phospho-caspase-3 antibody. Consistent with the TUNEL staining and FAS expression results, caspase-3 activation increased as the number of combined agents increased, with the WKYMVm+5-FU+mDCs triple combination inducing a dramatic increase in caspase-3 activity (Fig. 2). The levels of tumor cell apoptosis and caspase-3 activity correlate well with the tumor volume data in Fig. 1B.

### Administration of the triple combination causes the recruitment of CD8 T cells and NK cells into the tumor

**Figure 3:**
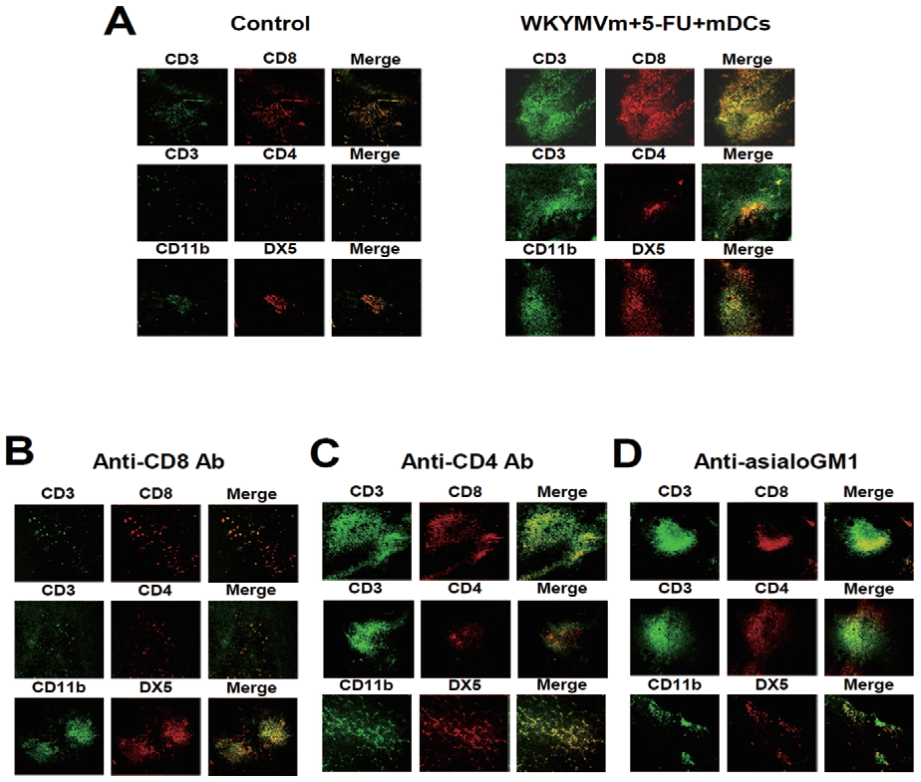
Triple administration of WKYMVm, 5-FU, and mDCs causes the recruitment of CD8 T lymphocytes, CD4 T lymphocytes, and NK cells in a heterotopic cancer animal model. (A) Balb/c mice were inoculated with CT-26 cells and treated with WKYMVm, 5-FU, and mDCs according to the protocol in Fig. 1A. CD8 T cells, CD4 T cells or NK cells were depleted by i.p. injection of 100 µg of anti-CD8 (B), anti-CD4 (C) or anti-asialoGM1 antibody (D) into Balb/c mice. Mice were euthanized 42 days after tumor inoculation and tumors were surgically excised, and processed for immunostaining using anti-CD3, anti-CD4, anti-CD8, and anti-asialoGM1 antibodies, as described in Materials and Methods. CD3^+^ CD8^+^ cells, CD3^+^ CD4^+^cells, and CD11b^+^ DX5^+^ cells indicate CD8 T lymphocytes, CD4 T lymphocytes, and NK cells, respectively. The data presented is representative of three independent experiments.

To initiate an anti-tumor immune response, leukocytic cells need to be recruited into the tumor area [18]. Since administration of the triple combination (WKYMVm+5-FU+mDCs) elicited potent anti-tumor activity against the heterotopic cancer animal model, we examined what types of cells are recruited into the tumor by immunohistochemistry using anti-CD3, anti-CD4, anti-CD8, anti-CD11b, or anti-DX5 antibody. Administration of the triple combination caused dramatic recruitment of CD8 T lymphocytes and NK cells into the tumor area, as well as slight recruitment of CD4 T lymphocytes (Fig. 3A).

Administration of anti-CD8 monoclonal antibody prior to the triple combination caused dramatic inhibition of CD8 T lymphocyte recruitment into the tumor area, without affecting NK cell recruitment (Fig. 3B). When anti-asialoGM1 antibody was administered prior to injection of the triple combination, NK cell recruitment into the tumor area was strongly blocked, but CD8 T lymphocyte recruitment was not affected (Fig. 3D). Anti-CD4 antibody administration prior to injection of the triple combination did not affect CD8 T lymphocyte or NK cell recruitment (Fig. 3C).

### CD8 T cells and NK cells play major roles in the anti-tumor activity of the triple combination therapy

**Figure 4:**
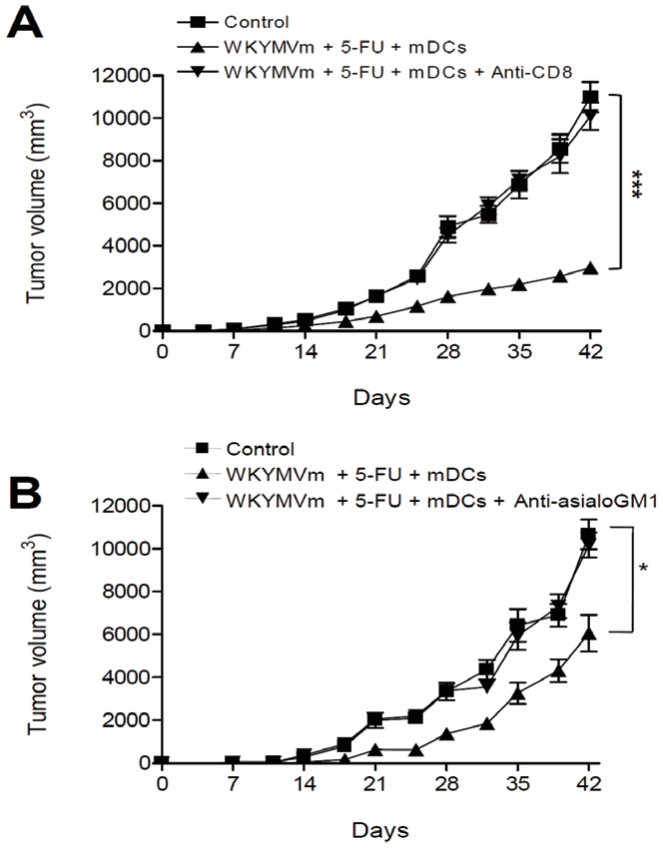
CD8 T lymphocytes and NK cells are involved in the anti-tumor activity induced by the combination of WKYMVm, 5-FU, and mDCs in a heterotopic cancer animal model. Balb/c mice were inoculated with CT-26 cells and treated with WKYMVm, 5-FU, and mDCs according to the protocol in Fig. 1A. CD8 T cells or NK cells were depleted by i.p. injection of 100 µg of anti-CD8 (A) or anti-asialoGM1 antibody (B) into Balb/c mice. Tumor volume was measured and the data are shown as the mean ± SEM (n = 8). ^*^, *P*<0.05, ^***^, *P*<0.001 compared with the control.

For efficient activation of anti-cancer activity in an experimental animal, several leukocyte types communicate with each other and act concertedly. We examined the relative contribution of individual leukocyte types on the anti-tumor activity induced by the triple combination therapy, by administrating antibodies against each leukocyte. When anti-CD8 antibody was administered to the heterotopic cancer animal model, the anti-tumor activity of the triple combination was almost completely inhibited (Fig. 4A). Administration of anti-asialoGM1 antibody elicited partial inhibition of the anti-tumor activity of the triple combination therapy (Fig. 4B). However, administration of the anti-CD4 antibody did not affect the anti-tumor activity of the triple combination (data not shown). These results indicate that CD8 T cells and NK cells play major roles in the anti-tumor activity of the triple combination of WKYMVm, 5-FU, and mDCs.

### Administration of the WKYMVm, 5-FU, and mDCs triple combination elicits a change of cytokine profile in a heterotopic cancer animal model

**Figure 5:**
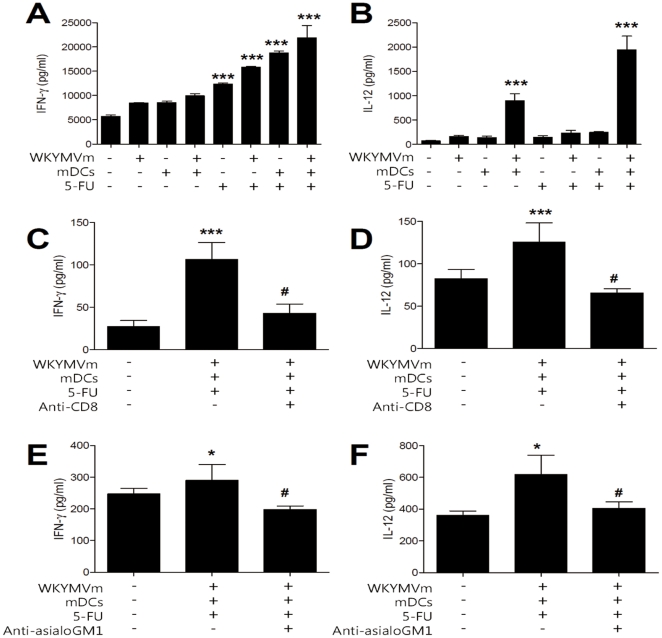
Effects of WKYMVm, 5-FU, and mDCs on IFN-γ and IL-12 production in an experimental animal model. Balb/c mice were inoculated with CT-26 cells and treated with WKYMVm, 5-FU, and mDCs according to the protocol in Fig. 1A. (A, B) IFN-γ and IL-12 levels were measured from tumor tissue lysate. (C–F) IFN-γ and IL-12 levels in peripheral blood upon triple combination treatment were measured. 100 µg of anti-CD8 (C, D) or anti-asialoGM1 antibody (E, F) was injected i.p. to Balb/c mice. Data are shown as the mean ± SEM (n = 8). ^*^, *P*<0.05, ^***^, *P*<0.001, compared with the control value; ^#^, *P*<0.05, significantly different from the WKYMVm+5-FU+mDCs-treated control.

We tested whether administration of WKYMVm, 5-FU, and mDCs in various combinations causes changes in cytokine profile compared to the control tumor lysate. As shown in Fig. 5A, administration of the triple combination caused dramatic changes in the levels of IFN-γ and IL-12 produced from tumor lysates in the heterotopic cancer animal model. The production of IFN-γ in the tumor gradually increased as the number of combined agents increased, with double combinations generally showing more IFN-γ production than single administration, and the triple combination showing the highest level of IFN-γ production (Fig. 5A). When IL-12 levels were measured, we found that single administration of each agent did not induce production of IL-12, and among the double combinations, only the combination of WKYMVm+mDCs enhanced IL-12 production in the animal model (Fig. 5B). Administration of the triple combination (WKYMVm+mDCs+5-FU) dramatically increased IL-12 production to a level that was two-fold higher than the WKYMVm+mDCs double combination (Fig. 5B).

Administration of the triple combination was also observed to enhance the production of IFN-γ and IL-12 in the peripheral blood (Fig. 5C–5F). To examine the role of CD8 T lymphocytes and NK cells in the production of these two cytokines upon triple combination administration, anti-CD8 or anti-asialoGM1 antibody was administered prior to triple combination treatment. Administration of anti-CD8 or anti-asialoGM1 antibody blocked triple combination-induced IFN-γ and IL-12 production in the peripheral blood (Fig. 5C–5F). These results indicate that CD8 T lymphocytes and NK cells play a role in IFN-γ and IL-12 production induced by triple combination treatment.

### Anti-metastasis activity of combined administration of WKYMVm, 5-FU, and mDCs

**Figure 6:**
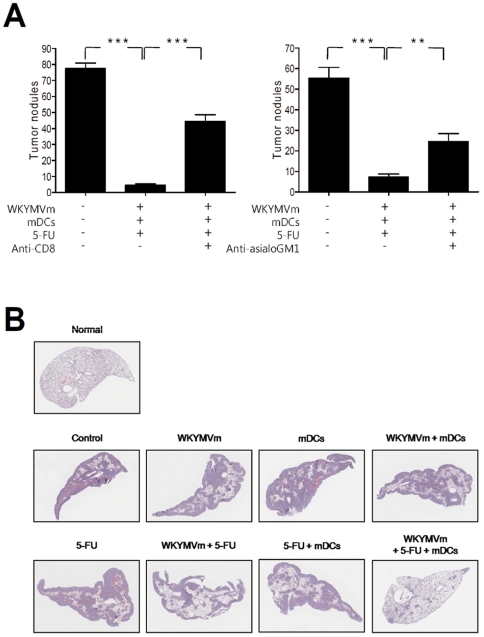
Anti-metastasis activity of WKYMVm, 5-FU, and mDCs. (A) Balb/c mice were inoculated with CT-26 cells and treated with WKYMVm, 5-FU, and mDCs according to the protocol in Fig. 1A. CD8 T cells or NK cells were depleted by injecting 100 µg of anti-CD8 or anti-asialoGM1 antibody i.p. into Balb/c mice. On day 42, mice were sacrificed and tumor nodules on the surface of the lungs were observed. (B) CT-26 cells (2×10^5^ cells in 100 µl of PBS) were injected into the tail vein of Balb/c mice. After 14 days, mice were sacrificed and hematoxylin and eosin staining was performed using the isolated lungs. The images shown are representative of eight independent experiments. ^**^, *P*<0.01, ^**^, *P*<0.01 compared with the control.

When developing an effective anti-tumor therapeutic agent, it is important to develop agents which inhibit tumor recurrence [19]. The metastatic ability of cancer cells to migrate from their origin to other target tissues is one of the major reasons that make it difficult to develop an efficient anti-cancer agent [20], [21]. In our heterotopic cancer animal model, we observed that subcutaneous inoculation of CT-26 colon cancer cells caused spontaneous metastasis of the cells into the lung tissue (Fig. 6A). Administration of the triple WKYMVm+mDCs+5-FU combination almost completely blocked metastasis of colon cancer cells to the lungs (Fig. 6A). This triple combination-induced anti-metastasis activity was blocked by the administration of anti-CD8 or anti-asialoGM1 antibody (Fig. 6A), indicating that the anti-metastasis activity is mediated by CD8 T lymphocyte and NK cell activity.

We also used an artificial metastasis animal model to test the effect of different combinations of WKYMVm, 5-FU, and mDCs against metastasis. Injection of CT-26 colon cancer cells into the tail vein causes metastasis into the lungs. Lungs were isolated 14 days after CT-26 cell injection. Dramatic lung metastasis was observed with vehicle treated mice (Fig. 6B). Single or double combinations of WKYMVm, mDCs, and 5-FU inhibited tumor metastasis to varying degrees to reduce lung metastasis, and the most potent effect was observed with the triple combination (Fig. 6B).

### Combined administration of WKYMVm, 5-FU, and mDCs enhances survival rates in a heterotopic cancer animal model

**Figure 7:**
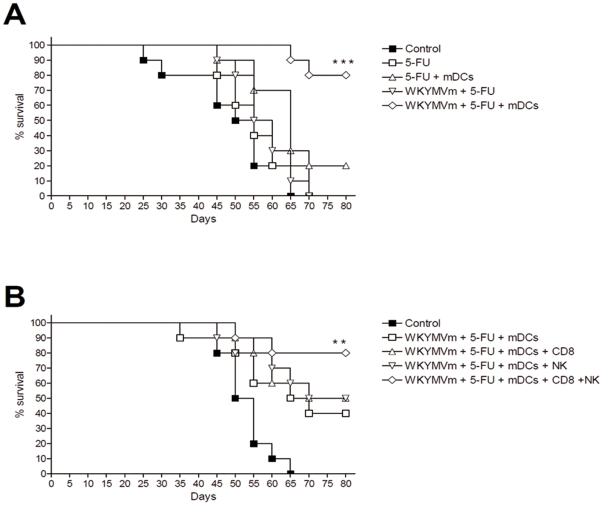
Enhancement of survival rate by WKYMVm, 5-FU, and mDCs in a heterotopic cancer animal model. (A) Survival rate of mice treated with combinations of WKYMVm, 5-FU, and mDCs. Balb/c mice were inoculated with CT-26 cells (5×10^5^ cells/head) and treated with WKYMVm, 5-FU, and mDCs according to the protocol in Fig. 1A. (B) Adoptive transfer of CD8 T lymphocytes or NK cells can enhance the survival rate of triple combination-treated heterotopic cancer animal model mice. Balb/c mice were inoculated with CT-26 cells (1×10^6^ cells/head) and treated with WKYMVm, 5-FU, and mDCs according to the protocol in Fig. 1A, and isolated CD8 T lymphocytes (1×10^6^ cells/head) or NK cells (1×10^6^ cells/head) were transferred into the tail vein. Survival rate was monitored for 80 days. ^**^, *P*<0.01, ^***^, *P*<0.001 compared with the control.

The survival rate of the heterotopic cancer animal model upon combined treatment was examined. We tested the combinations that showed relatively high anti-tumor activity in our previous assays above; 5-FU alone, 5-FU+WKYMVm, 5-FU+mDCs, and 5-FU+mDCs+WKYMVm. Administration of 5-FU alone, 5-FU+mDCs, or 5-FU+WKYMVm only slightly increased survival rate, but administration of the triple 5-FU+mDCs+WKYMVm combination strongly enhanced survival rate (Fig. 7A). All mice in the control group were died after 65 days, whereas 80% of the triple combination-administered group was alive at 80 days (Fig. 7A).

To test if adoptive transfer of CD8 T lymphocytes and NK cells could enhance the triple combination-induced survival of the heterotopic cancer animal model mice, we inoculated an increased number of colon cancer cells (1×10^6^ cells/head) into mice. In this model, the effect of triple combination was decreased, reducing the survival rate to 40% at 80 days (Fig. 7B). Under these conditions adoptive transfer of CD8 T lymphocytes or NK cells was performed. Adoptive transfer of CD8 T lymphocytes or NK cells only slightly increased the survival rated caused by triple combination administration, whereas combined adoptive transfer of CD8 T lymphocytes and NK cells dramatically increased survival rate (Fig. 7B). These results indicate that both CD8 T lymphocytes and NK cells can enhance the survival rate induced by the triple combination in the heterotopic cancer animal model.

## Discussion

In this study, we demonstrated that administration of the triple combination of the synthetic peptide WKYMVm, mDCs, and 5-FU endows potent anti-cancer immunity. Single (WKYMVm, mDCs, or 5-FU) or double (WKYMVm+mDCs, 5-FU+WKYMVm or 5-FU+mDCs) treatment of mice slightly decreased tumor volume, but administration of the triple combination dramatically inhibited tumor growth (Fig. 1). The tumor volume measurement results were correlated well with FAS expression, caspase-3 activity, and TUNEL staining results (Figs. 1 and 2). According to previous reports, IFN-γ is known to induce the expression of cell death receptors such as FAS [22], [23]. In this study, we showed that the administration of different combinations of WKYMVm, 5-FU, or mDCs can induce IFN-γ expression, with the triple combination causing the most enhanced IFN-γ production (Fig. 5A). The expression level of IFN-γ was well correlated with the anti-tumor activity of each combination, suggesting that IFN-γ induced by the administration of each agent or combination is critically involved in anti-tumor activity in the animal model.

IL-12 can prime or stimulate CD4 T cells, CD8 T cells and NK cells [24], [25], [26]. In this study, only certain combinations, namely WKYMVm+mDCs and the triple combination could induce IL-12 expression in the heterotopic cancer animal model (Fig. 5B). Single administration of WKYMVm or mDCs did not induce IL-12 production (Fig. 5B). This suggests that the stimulation of mDCs with WKYMVm is responsible for efficient induction of IL-12 in the animal model. Since WKYMVm is a ligand for the FPR family, it appears likely that the activation of the FPR family induces IL-12 expression from mDCs. In the survival rate experiment, triple administration of WKYMVm+5-FU+mDCs induced a strong enhancement of survival rate in a heterotopic cancer animal model, but 5-FU+mDCs treatment had a much lesser effect (Fig. 7). Since IL-12 production is strongly induced by the triple combination but not by 5-FU+mDCs (Fig. 5B), it may be possible that the triple combination enhances survival rate by stimulating the production of soluble factors such as IL-12.

NK and CD8 T cells were recently reported to be important in cancer regression [27], [28], [29]. Here, we demonstrated that CD8 T and NK cells were enriched in tumors when the triple combination was administered (Fig. 3), and depletion of CD8 T cells or NK cells increased tumor growth (Fig. 4). In addition, depletion of CD8 T cells or NK cells reduced the levels of induced IFN-γ and IL-12 (Fig. 5C–5F). These results indicate that CD8 T cells and NK cells are involved in the anti-tumor effect of the triple combination. Triple administration of WKYMVm, mDCs and 5-FU also elicited an anti-metastasis effect in a heterotopic cancer animal model (Fig. 6). Intravenous injection of ATRA-cationic liposome/IL-12 pDNA complexes was shown to enhance the growth inhibition of metastatic lung tumors in mice [30], supporting our notion that IL-12 production may be associated with anti-metastasis activity of the triple combination.

In this study we demonstrated that CD8 T cells and NK cells play important roles in the anti-tumor activity of the triple combination. Previously we reported that NK cells express functional FPR family, and stimulation of NK cells with FPR agonist WKYMVm elicits IFN-γ production [11]. In this study we also found that mouse NK cells express FPR family, and stimulation of mouse NK cells with WKYMVm elicits the production of IFN-γ (data not shown). This finding explains WKYMVm-induced NK cell activation is essentially required for the anti-tumor activity of the triple combination against heterotopic cancer animal model. In terms of the activation of CD8 T cells and their role in the heterotopic cancer animal model, previously we showed that T cells do not express receptors for WKYMVm [31]. Since CD8 T cells can be activated by mDCs and IL-12 [32], [33], it will be reasonable to think that the administration of triple combination containing WKYMVm can induce the activation of CD8 T cell will be mediated by mDCs or IL-12.

In conclusion, triple combination immunotherapy with mDCs, the synthetic peptide WKYMVm, and 5-FU was superior to single or double treatment in inhibiting established primary tumors and metastasis, as well as prolonging survival. The increase in therapeutic efficacy was due to effects that occurred locally (enhanced levels of FAS and caspase-3 expression) and systemically (increased production of IFN-γ and IL-12). Therefore, use of the triple combination therapy may have implications in solid tumor and metastasis treatment.

## Materials and Methods

### Mice

The Institutional Review Committee for Animal Care and Use at Dong-A University specifically approved this study (approval ID: DIACUC 07-6). Balb/c mice (males, 6–8 weeks old) were obtained from the Jackson Laboratory.

### Cell culture and material

CT-26 cells (CRL-2638; ATCC American Type Culture Collection) are a colon adenocarcinoma cell line derived from Balb/c mice. CT-26 cells were maintained in RPMI 1640 (Invitrogen, Carlsbad, CA) containing 10% heat-inactivated fetal bovine serum and antibiotics at 37°C in a 5% CO_2_ humidified incubator. The synthetic peptide, WKYMVm, was synthesized at Anygen (Kwangju, Korea). The purity of synthesized WKYMVm was >98%. 5-FU was purchased from Choongwae, Pharma Co. (Seoul, Korea).

### DC preparation and maturation

Bone marrow cells were cultured for 5 days in DC medium (RPMI 1640 medium with 10% FBS, 2 mM L-glutamine, 50 µM β-mercaptoethanol, antibiotics) in the presence of mouse GM-CSF (1000 U/ml) and IL-4 (500 U/ml), as described [34]. GM-CSF and IL-4 were replenished on day 2 and 4. On day 5, cells were collected and transferred onto a new plate with DC medium and stimulated with lipopolysaccharide (100 ng/ml; Sigma), CpG oligodeoxynucleotide 1826 (10 µg/ml), and CT-26 lysate (100 µg/ml) for 2 days to induce maturation. On day 7, DCs were collected for use as vaccines.

### Tumor growth and survival

To measure tumor growth, CT-26 cells (5×10^5^ cells in 100 µl of PBS) were injected s.c. into the right flank of Balb/c mice (n = 8) on day −3. On days 0 and 1, 5-FU (100 µg/100 µl) was injected s.c. into Balb/c mice. On day 2, mice were treated with four injections of WKYMVm (100 µg/100 µl) and mDCs (1×10^6^ cells) at 12 h-intervals. On days 4 and 5, 5-FU (100 µg/100 µl) was injected s.c. into Balb/c mice. Subsequently, the mice were treated with s.c. injection of 5-FU, mDCs and WKYMVm once weekly for 4 weeks. Tumor volume was determined by the following formula: tumor volumes (in mm^3^) = length (mm)×width (mm)^2^/2 [35]. In addition to monitoring tumor growth, mice were observed for survival following tumor inoculation and treatment.

### Hematoxylin and eosin and immunofluorescence staining

Mice were euthanized 42 days after tumor inoculation, and tumors were surgically excised, fixed for 24 h in 10% neutral phosphate buffered formalin (NBF), embedded in paraffin, and sectioned and stained with hematoxylin and eosin for morphological analysis. For immunostaining, the following primary antibodies were used: anti- mouse Fas (Santa Cruz), anti-mouse cleaved caspase-3 (Cell Signaling), FITC-conjugated anti-mouse CD3 (BD Pharmingen), PE-conjugated anti-mouse CD4 (BD Pharmingen), PE-conjugated anti-mouse CD8 (BD Pharmingen), FITC-conjugated anti- mouse CD11b (BD Pharmingen), and PE-conjugated anti- mouse DX5 (BD Pharmingen) antibody. For confocal microscopy, fixed tumor tissues were stained with FITC-conjugated anti-mouse IgG and Alexa 594-conjugated anti-mouse IgG (BD Pharmingen).

### *In situ* TUNEL staining

Terminal deoxy-nucleotidyl transferase-mediated digoxigenin-dUTP nick end labeling (TUNEL) was performed to detect apoptotic cells in the tumor tissues. Paraffin sections were deparaffinized, hydrated, treated with 3% H_2_O_2_ for 5 min, and rinsed with PBS for 15 min, and the *In situ* Death Detection Kit, POD, was used (Roche, Penzberg, Germany). Briefly, digoxigenin-dUTP end-labeled DNA was detected with anti-digoxigenin-peroxidase antibody followed by peroxidase detection with diaminobenzidine (DAB). Tissues were counterstained with Mayer's hematoxylin.

### Enzyme-linked immunosorbent assay (ELISA)

On day 42, blood was collected from the heart of mice and clarified by centrifugation. The serum was stored at −80°C until ready for cytokine analysis. Murine IL-12 (p70) and IFN-γ concentrations were measured using a standardized sandwich ELISA method (BD Biosciences Pharmingen).

### *In vivo* immune cell subset depletion

To deplete CD4, CD8 T cells and NK cells, mice were treated with the corresponding antibody on days −1, 0, and 5 (where day 0 is the day of primary tumor inoculation). The monoclonal rat anti-mouse CD4 (clone GK1.5), rat anti-mouse CD8 (clone 53-6.7) and rat anti-mouse asialoGM1 antibodies were used for immunodepletion. In the leukocyte depletion studies, 100 µg of anti-CD4, anti-CD8, or anti-asialoGM1 antibody was injected i.p. into Balb/c mice.

### Lung metastasis

For the heterotopic lung metastasis experiment, CT-26 cells (5×10^5^ cells in 100 µl of PBS) were injected s.c. into the right flank of Balb/c mice (n = 8) on day −3. 5-FU, WKYMVm, and mDCs were administered for 3 weeks, as described above for the experiment to measure tumor volume. On day 42, mice were sacrificed and tumor nodules on the surface of the lungs were observed. For the metastatic cancer animal experiment, CT-26 cells were washed twice with PBS, and injected into the tail vein of Balb/c mice (2×10^5^ cells in 100 µl of PBS). After 14 days, mice were sacrificed and the lung was weighed.

### Adoptive transfer

To obtain CT26 tumor-reactive CD8 T cells, Balb/c mice were immunized three times (at 1-week intervals) with 1×10^7^ CT26 tumor lysates (s.c. injection). On 21 day, immunized mice were sacrificed, and CD8 T cells or NK cells were isolated using CD8 T cell isolation kit or NK cell isolation kit (Miltenyi Biotec GmbH, Bergisch Gladbach, Germany) from their spleen. For adoptive therapy, tumors were established by s.c. injecting 5×10^6^ CT26 tumor cells on the right flank of Balb/c mice. Palpable tumors (∼5 mm in diameter) usually formed at the injection site after 4 to 5 days. Before triple combined treatment, purified CD8 T (1×10^6^ cell/100 µl) cells or NK (1×10^6^ cell/100 µl) cells transferred (i.v. injection) to CT26 tumor-loaded mice and then begin with triple combined treatment.

### Data analysis

Results are presented as the mean ± SEM of repeated experiments. Statistical significance of differences was determined using ANOVA. Survival data were analyzed using the log-rank test. *P*<0.05 was considered statistically significant.
